# Disruption of mitochondrial complex III in cap mesenchyme but not in ureteric progenitors results in defective nephrogenesis associated with amino acid deficiency

**DOI:** 10.1016/j.kint.2022.02.030

**Published:** 2022-03-24

**Authors:** Nan Guan, Hanako Kobayashi, Ken Ishii, Olena Davidoff, Feng Sha, Talat A. Ikizler, Chuan-Ming Hao, Navdeep S. Chandel, Volker H. Haase

**Affiliations:** 1 Department of Medicine, Vanderbilt University Medical Center and Vanderbilt University School of Medicine, Nashville, Tennessee, USA; 2 Division of Nephrology, Huashan Hospital and Nephrology Research Institute, Fudan University, Shanghai, China; 3 The Vanderbilt O’Brien Kidney Center, Vanderbilt University School of Medicine, Nashville, Tennessee, USA; 4 Department of Medicine, Feinberg School of Medicine, Northwestern University Chicago, Illinois, USA; 5 Department of Molecular Physiology and Biophysics, Vanderbilt University School of Medicine, Nashville, Tennessee, USA; 6 Section of Integrative Physiology, Department of Medical Cell Biology, Uppsala University, Uppsala, Sweden

**Keywords:** amino acids, kidney development, mitochondria, mitochondrial complex III, mitochondrial electron transport chain, TCA cycle

## Abstract

Oxidative metabolism in mitochondria regulates cellular differentiation and gene expression through intermediary metabolites and reactive oxygen species. Its role in kidney development and pathogenesis is not completely understood. Here we inactivated ubiquinone-binding protein QPC, a subunit of mitochondrial complex III, in two types of kidney progenitor cells to investigate the role of mitochondrial electron transport in kidney homeostasis. Inactivation of QPC in sine oculis-related homeobox 2 (SIX2)–expressing cap mesenchyme progenitors, which give rise to podocytes and all nephron segments except collecting ducts, resulted in perinatal death from severe kidney dysplasia. This was characterized by decreased proliferation of SIX2 progenitors and their failure to differentiate into kidney epithelium. QPC inactivation in cap mesenchyme progenitors induced activating transcription factor 4–mediated nutritional stress responses and was associated with a reduction in kidney tricarboxylic acid cycle metabolites and amino acid levels, which negatively impacted purine and pyrimidine synthesis. In contrast, QPC inactivation in ureteric tree epithelial cells, which give rise to the kidney collecting system, did not inhibit ureteric differentiation, and resulted in the development of functional kidneys that were smaller in size. Thus, our data demonstrate that mitochondrial oxidative metabolism is critical for the formation of cap mesenchyme-derived nephron segments but dispensable for formation of the kidney collecting system. Hence, our studies reveal compartment-specific needs for metabolic reprogramming during kidney development.

A major function of mitochondria in the kidney is to provide adenosine triphosphate (ATP) for energy-consuming transport functions in tubular epithelial cells.^1^ Mitochondria also operate as signaling organelles and regulate gene expression and cellular differentiation via tricarboxylic acid (TCA) cycle metabolites and reactive oxygen species (ROS), both of which are interconnected with mitochondrial (mt) electron transport.^2^

The mt electron transport chain consists of 4 large multisubunit enzyme complexes that transfer electrons from donors, such as reduced nicotinamide adenine dinucleotide (NADH) and succinate, to molecular oxygen. Mt complex I (NADH: ubiquinone oxidoreductase) oxidizes NADH, mt complex II (succinate-coenzyme Q reductase) catalyzes the oxidation of succinate to fumarate, and mt complex III transfers high-energy electrons from the ubiquinol form of coenzyme Q10 to cytochrome c. Cytochrome c is then oxidized by mt complex IV, the terminal electron-accepting complex that reduces O_2_ to H_2_O. During electron transfer, protons are pumped into the mt intermembrane space and generate an electrochemical gradient that drives ATP synthesis through chemiosmosis.^3^

The quinol (Q) cycle carries out the sequential oxidation and reduction of the electron carrier coenzyme Q10 within mt complex III. Inactivation of mt complex III results in the interruption of mt electron transport, the inhibition of oxidative phosphorylation, and mt ATP production and changes the cellular redox state by decreasing cellular NAD^+^ levels.^4^ The degree by which alterations in mt electron transport and shifts in oxidative metabolism impact on kidney development and physiology or the pathogenesis of kidney diseases is not well understood.

To examine the role of mt electron transport in kidney development and homeostasis, we genetically inactivated subunit VII (UQCRQ) of the mt ubiquinol-cytochrome c reductase complex III, also known as Q-binding protein (QPC), in sine oculis–related homeobox 2 (SIX2)–expressing cap mesenchyme (CM) cells or in homeobox B7–expressing ureteric bud (UB) epithelium. SIX2-expressing progenitor cells give rise to podocytes and all nephron segments except for the collecting duct (CD),^5^ whereas homeobox B7 is expressed in the Wolffian duct, the UB, and their derivatives during excretory system development.^6,7^ CM and UB interact reciprocally, as the CM produces trophic factors that induce branching of the adjacent UB, such as glial cell line–derived neurotrophic factor and fibroblast growth factor 10, whereas the UB produces factors such as fibroblast growth factors and wingless-type MMTV integration site family, member 9B (WNT9B), that expand and/or maintain CM progenitors and promote their differentiation through mesenchymal to epithelial transition (MET).^5,8–11^ Comprehensive analyses of these interactions on a cellular, molecular, and metabolic level are not only critical for understanding processes that underlie kidney injury and repair, but also for developing strategies to recapitulate nephrogenesis for therapeutic purposes.^12^

Here, we report that disruption of mt complex III electron transport affected the developing kidney differentially. Whereas mt electron transport was dispensable for epithelial morphogenesis of the ureteric tree, loss of mt electron transport in SIX2 progenitors resulted in severe kidney dysplasia associated with reduced proliferation and failure to undergo MET. Disruption of mt electron transport in SIX2 progenitors was furthermore associated with a reduction in kidney TCA cycle metabolites and kidney amino acid levels. Our data establish compartment-specific needs for metabolic reprogramming in the developing kidney.

## METHODS

### Mouse strains

The generation and genotyping of mice carrying floxed *Uqcrq* (*Qpc*) alleles has been described elsewhere.^13^ For the inactivation of *Qpc* in CM progenitor cells, mice homozygous for the *Qpc* floxed allele were bred to bacterial artificial chromosome transgenic mice that express Cre recombinase fused to enhanced green fluorescent protein (eGFP) under the control of the *Six2* promoter,^14^ generating *Six2-eGFP/cre*^*tg/+*^;*Qpc*^*fox/flox*^ mice, referred to as *Six2-Qpc*^−/−^ mutants. For the inactivation of QPC in UB epithelium, *Hoxb7-cre* transgenics were used,^15^ generating *Hoxb7-cre*^*tg*/+^;*Qpc*^*floxl/flox*^ mice, referred to as *Hoxb7-Qpc*^−/−^ mutants. To monitor *Six2-cre* or *Hoxb7-cre* activity, we took advantage of the *ROSA26-ACTB-tdTomato,-EGFP* double-fluorescent Cre reporter allele, referred to as *mT/mG*.^16^ All procedures involving mice were performed in accordance with National Institutes of Health guidelines for the use and for care of live animals and were approved by the Vanderbilt University Institutional Animal Care and Use Committee.

### Statistical analysis

Data are reported as mean ± SEM. Statistical analyses were performed with Prism 6 software (GraphPad Software Inc.) using the Student *t* test. *P* values of less than 0.05 were considered statistically significant.

### Supplemental methods

Detailed information regarding DNA, RNA, protein, morphologic, and metabolic analyses can be found in Supplementary Materials and Methods. Primer sequences are listed in Supplementary Table S1, and primary antibodies are listed in Supplementary Table S2.

## RESULTS

### Disruption of mt electron transport in SIX2 progenitors results in severe kidney dysplasia

**Figure 1 |:**
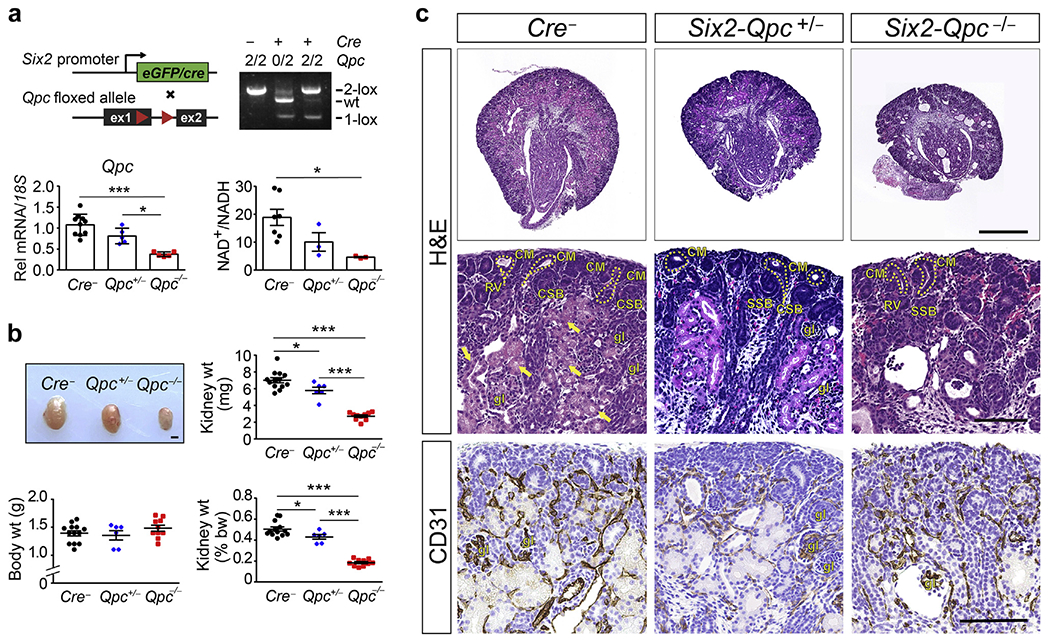
Conditional inactivation of mitochondrial complex III subunit QPC in SIX2 nephron progenitor cells results in severe kidney dysplasia. (**a**) Upper panels: schematic illustrating the experimental approach and location of targeted sequences within the conditional *Qpc* allele; loxP sites are depicted by red arrows. Polymerase chain reaction (PCR) analysis of total genomic DNA isolated from *Cre*^−^ littermate controls, *Six2-Qpc*^+/−^ and *Six2-Qpc*^−/−^ kidneys at postnatal day (P) 0. The genotype of mice is indicated; the number 2 represents the 2-lox non-recombined allele, and the plus and minus signs indicate the presence or absence of the *Cre* transgene. Lower-left panel: *Qpc* transcript levels in control, *Six2-Qpc*^+/−^ and *Six2-Qpc*^−/−^ kidneys at age P0 analyzed by quantitative real-time PCR (n = 5–6). Lower-right panel: NAD+/NADH ratio in whole kidney tissues (n = 3–5). (**b**) Macroscopic images of kidneys from *Cre*^−^ littermate control, *Six2-Qpc*^+/−^ and *Six2-Qpc*^−/−^ mutants at age P0. Bar = 1 mm. Kidney weight (wt), body weight, and kidney/body weight ratio at P0 (n = 8). (**c**) Representative images of kidney sections from control, *Six2-Qpc*^+/−^ and *Six2-Qpc*^−/−^ mice at age P0 stained with hematoxylin and eosin (H&E) and immunohistochemistry (IHC) for cluster of differentiation 31 antigen (CD31). Ureteric buds are outlined by dashed lines. Bar = 500 μm for the top panel and 100 μm for H&E stains and IHC sections. Data are presented as mean ± SEM; Student’s *t* test, 2-tailed, *P < 0.05, **P < 0.01, and ***P < 0.001. *18S,* 18S ribosomal RNA; CM, cap mesenchyme; CSB, comma-shaped body; gl, glomerulus; NAD, nicotinamide adenine dinucleotide; NADH, reduced nicotinamide adenine dinucleotide; Rel, relative; RV, renal vesicle; SSB, S-shaped body. To optimize viewing of this image, please see the online version of this article at www.kidney-international.org.

To examine the role of mt electron transport in nephron development and function, we inactivated mt complex III subunit QPC in SIX2-expressing progenitor cells (Figure 1a). Recombination efficiency was assessed by genomic polymerase chain reaction and analysis of *Qpc* transcript levels in kidney tissue. Both assays suggested efficient targeting of the floxed *Qpc* allele (Figure 1a). A significant decrease in tissue NAD^+^/NADH ratios was consistent with inactivation of mt electron transport (Figure 1a).

**Figure 2 |:**
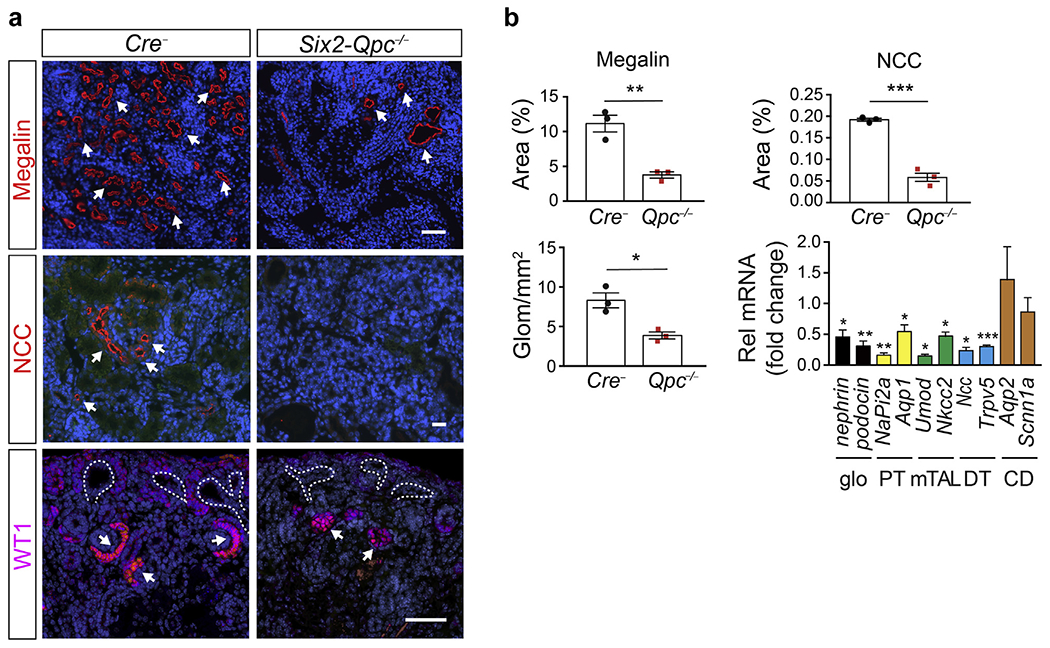
Nephron formation is defective in *Six2-Qpc*^−/−^ mutant mice. (**a**) Shown are representative immunofluorescence images of formalin-fixed, paraffin-embedded kidney sections from *Cre*^−^ littermate control and *Six2-Qpc*^−/−^ mice at age postnatal day (P) 0, stained for megalin, thiazide-sensitive sodium chloride cotransporter (NCC), and Wilms tumor 1 protein (WT1). Ureteric buds are outlined by dashed white lines. The white arrows identify positive staining. Bar = 50 −m. (**b**) Quantification of megalin-NCC–expressing nephron segments and glomeruli in control and *Six2-Qpc*^−/−^ mice. Shown is positively stained area or number of glomeruli (Glom) per mm^2^. Glomerular and nephron segment–specific gene expression in total kidney homogenates from *Six2-Qpc*^−/−^ mutants at age P0. Gene expression was compared with *Cre*^−^ littermate controls and expressed as fold change (n = 5 each). Data represent mean ± SEM; Student’s *t* test, 2-tailed, *P < 0.05, **P < 0.01, and ***P < 0.001. *Aqp1,* aquaporin 1; *Aqp2,* aquaporin 2; CD, collecting duct; DT, distal tubule; glo, glomerular; mTAL, medullary thick ascending loop of Henle; *NaPi2a*, sodium-phosphate cotransporter 2A; *Nkcc2,* sodium-potassium-chloride cotransporter 2; PT, proximal tubule; Rel, relative; *Scnn1a*, epithelial sodium channel 1 alpha subunit; *Trpv5,* transient receptor potential cation channel subfamily V member 5; *Umod*, uromodulin. To optimize viewing of this image, please see the online version of this article at www.kidney-international.org.

Although born at expected Mendelian frequencies and normal-appearing, *Six2-Qpc*^−/−^ pups died within 2 days after birth. Although newborn *Six2-Qpc*^−/−^ mice exhibited normal body weight, kidneys were significantly smaller compared with *Cre*^−^ littermate controls; kidney to body weight ratio of 0.19% ± 0.05% versus 0.40% ± 0.13% for controls; n = 8 each, *P* < 0.001 (Figure 1b). Histologic analysis of formalin-fixed, paraffin-embedded kidney sections revealed a severe reduction in the number of glomeruli and mature nephron structures (Figure 1c). Nephrogenic zone structures such as comma-shaped bodies and S-shaped bodies were easily identifiable by hematoxylin and eosin and toluidine blue staining in normal but not in mutant kidneys (Figure 1c and Supplementary Figure S1). In line with these findings were reduced expression levels of nephron segment markers megalin, thiazide-sensitive sodium chloride cotransporter, and Wilms’ tumor protein 1 analyzed by immunofluorescence (IF), and the absence of lotus tetragonolobus lectin binding (Figure 2a and Supplementary Figure S1). Transcript levels of nephrin, podocin, sodium-phosphate cotransporter-2a (*NaPi2a*), aquaporin 1 (*Aqp1*), uromodulin (*Umod*), sodium-potassium-chloride cotransporter 2 (*Nkcc2*), *Ncc*, and transient receptor potential cation channel subfamily V member 5 (*Trpv5*) were reduced at age postnatal day (P) 0 (Figure 2b) and embryonic day (E) 15.5 (Supplementary Figure S2). In contrast, transcript levels of CD markers aquaporin 2 (*Aqp2*) and epithelial sodium channel 1 alpha subunit (*Scnn1a*) were not significantly changed in *Six2-Qpc*^−/−^ kidneys (Figure 2b and Supplementary Figure S2). Despite a 47% reduction in the NAD^+^/NADH ratio at birth, mice with heterozygous *Qpc* deficiency (*Six2-Qpc*^+/−^) developed normally and were fertile (Figure 1). Kidney weight was reduced by approximately 17% at birth (Figure 1) and comparable to *Cre*^−^ littermate controls at 6 weeks of age (data not shown). Differences in blood urea nitrogen levels between 6-week-old *Six2-Qpc*^+/−^ heterozygotes and *Cre*^−^ littermate controls were not observed (data not shown). Taken together, these data demonstrate that disruption of mt electron transport in SIX2 progenitor cells resulted in severe kidney dysplasia characterized by absent nephron development.

### Disruption of mt electron transport in ureteric bud epithelium does not result in kidney dysplasia

**Figure 3 |:**
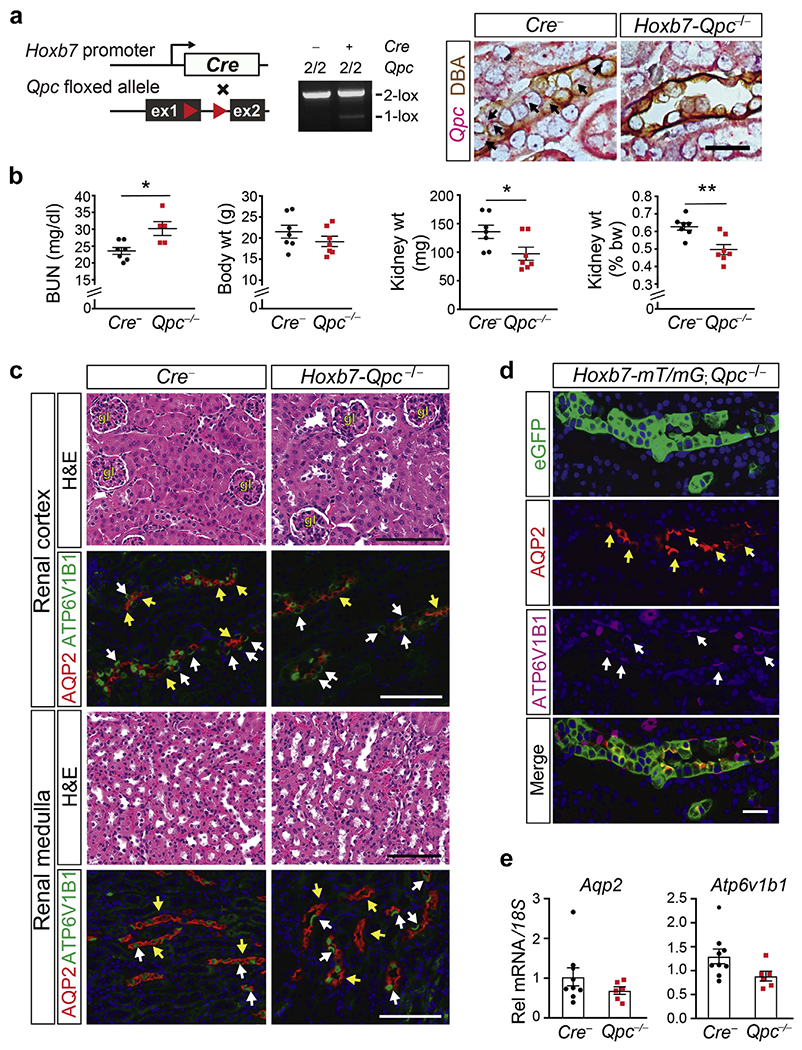
Mitochondrial electron transport is dispensable for ureteric epithelial differentiation. (**a**) Schematic illustrating experimental approach and location of targeted sequences within the floxed *Qpc* allele; loxP sites are depicted by red arrows. Polymerase chain reaction (PCR) analysis of total genomic DNA isolated from littermate control and *Hoxb7-Qpc*^−/−^ 6-week-old kidneys. The genotype of mice is indicated; the 2-lox non-recombined allele is represented by the number 2; plus and minus signs indicate the presence or absence of the *Cre* transgene. Right panel: *Qpc* mRNA detected by colorimetric RNA *in situ* hybridization in *Cre*^−^ littermate control and *Hoxb7-Qpc*^−/−^ kidneys. Kidney sections were costained with dolichos biflorus agglutinin (DBA) lectin; the black arrows depict *Qpc* transcripts (red signal). Bar = 25 μm. (**b**) Blood urea nitrogen (BUN), total body weight (wt), kidney weight, and kidney/body weight ratio for control and *Hoxb7-Qpc*^−/−^ mutants at age 6 weeks (n = 7). (**c**) Representative hematoxylin and eosin (H&E) and immunofluorescence (IF) images of kidney sections from 6-week-old control and *Hoxb7-Qpc*^−/−^ mice. IF staining for collecting duct markers aquaporin 2 (AQP2) and ATPase H^+^ transporting V1 subunit B1 (ATP6V1B1). The yellow arrows depict AQP2^+^ cells, and the white arrows identify ATP6V1B1^+^ cells. Bar = 100 μm. (**d**) Representative images of kidney sections from 6-week-old *Hoxb7-mT/mG;Qpc*^−/−^ mice stained by IF for enhanced green fluorescent protein (eGFP), AQP2, and ATP6V1B1; the yellow arrows depict AQP2^+^ cells, and the white arrows identify ATP6V1B1^+^ cells; the presence of eGFP indicates recombination of the Cre reporter allele (*mT/mG*). Bar = 25 μm. (**e**) Analysis of *Aqp2* and *Atp6v1b1* transcript levels in kidneys from 6-week-old *Cre*^−^ littermate control and *Hoxb7-Qpc*^−/−^ kidneys by quantitative real-time PCR (n = 6–8). Data are expressed as mean ± SEM; Student’s *t* test, 2-tailed, *P < 0.05, **P < 0.01. *18S,* 18S ribosomal RNA; Rel, relative. To optimize viewing of this image, please see the online version of this article at www.kidney-international.org.

To investigate the role of mt electron transport in the development of the kidney collecting system, we used *Hoxb7-cre* transgenic mice to induce robust *Qpc* inactivation along the entire UB epithelium and adult collecting system.^15^ In contrast to *Qpc* inactivation in SIX2 progenitor cells, *Qpc* deletion in UB did not result in premature lethality. The expression levels of kidney development and tubular differentiation markers were not different from *Cre*^−^ littermate controls in P0 kidneys (Supplementary Figure S4). *Hoxb7-Qpc*^−/−^ mutants developed normally into adulthood and were fertile. Analysis of Cre-mediated recombination by genomic polymerase chain reaction and RNA *in situ* hybridization (BaseScope) in conjunction with immunohistochemistry demonstrated that inactivation of *Qpc* was efficient in UB tips and trunk before birth (Supplementary Figure S3) and still detectable in CD epithelial cells (Base-Scope in conjunction with lectin histochemistry) at age 6 weeks (Figure 3a). *Hoxb7-Qpc*^−/−^ mutant mice were characterized by smaller kidneys at birth and at age 6 weeks (97.2 ± 30.6 mg vs. 135.7 ± 30.8 mg for controls at 6 weeks of age; n = 7, *P* = 0.037), whereas body weights were comparable to *Cre*^−^ littermate controls (Figure 3b and Supplementary Figure S4). Blood urea nitrogen levels were within the normal range and stable over time with 30.2 ± 4.6 mg/dl at 6 weeks and 29.7± 2.59 mg/dl at 6 months of age (n = 7 and 4, respectively; data not shown). Hematoxylin and eosin staining did not identify any major histologic abnormalities in the kidney cortex or medulla compared with *Cre*^−^ littermate controls at birth and at age 6 weeks (Figure 3c and Supplementary Figure S4).

We performed IF staining for AQP2 and ATPase H^+^ transporting V1 subunit B1 (ATP6V1B1). Similar expression patterns were found in *Hoxb7-Qpc*^−/−^ mutants and controls, suggesting that *Qpc* deficiency did not impair CD differentiation (Figure 3c). IF staining of kidney sections from *Hoxb7-mT/mG; Qpc*^−/−^ mice demonstrated that AQP2 or ATP6V1B1 was consistently coexpressed with eGFP, indicating that cells had undergone Cre-mediated recombination (Figure 3d). In line with these findings were whole kidney *Aqp2* and *Atp6v1b1* transcript levels in *Hoxb7-Qpc*^−/−^ kidneys, which were not different from controls (Figure 3e). Taken together, our data suggest that mt complex III activity and electron transport were dispensable for the formation of the kidney collecting system.

### Mitochondrial complex III subunit QPC is required for CM mesenchyme to epithelial transition

**Figure 4 |:**
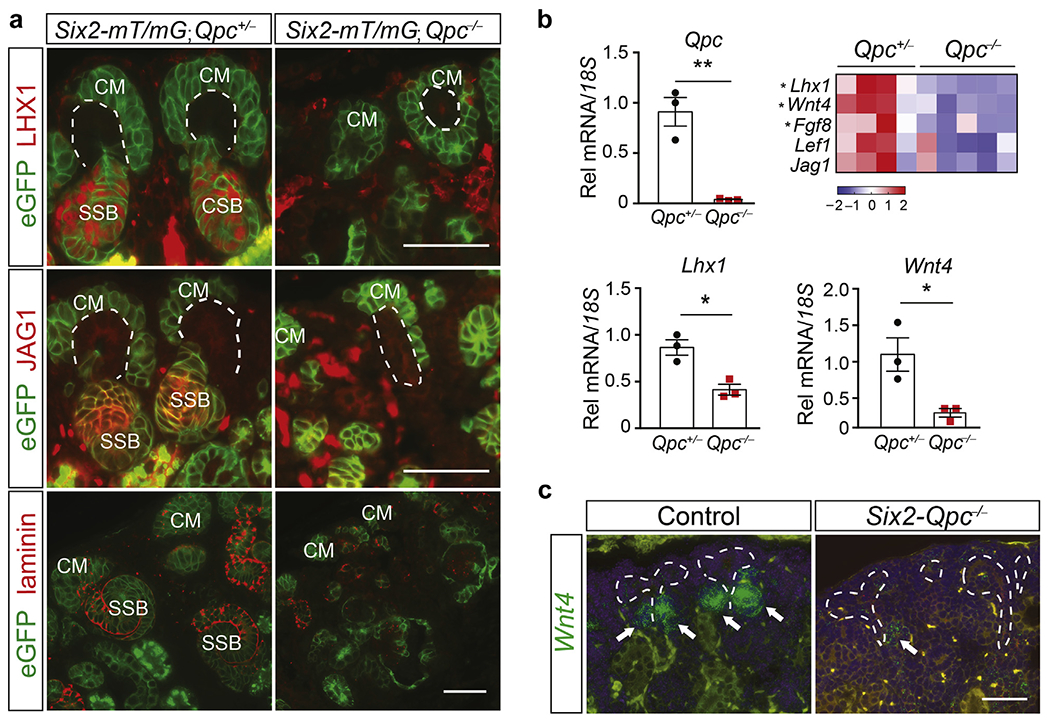
Inactivation of mitochondrial complex III subunit QPC in SIX2 nephron progenitors inhibits mesenchyme to epithelial transition. (*a*) Representative immunofluorescence images of kidney sections from *Six2-mT/mG;Qpc*^−/−^ and *Six2-mT/mG;Qpc*^−/−^ mice at age postnatal day (P) 0, costained for green fluorescent protein (eGFP) and LIM homeobox 1 (LHX1), Jagged 1 (JAG1), or laminin. eGFP expression indicates recombination of the Cre reporter allele *(mT/mG).* Ureteric buds are outlined by dashed white lines. Bar = 50 μm. (**b**) Expression levels of genes associated with mesenchymal epithelial transition and nascent nephron formation in *Six2-eGFP/cre*–expressing cells; cells were isolated by fluorescence-activated cell sorting from *Six2-Qpc*^+/−^ control and *Six2-Qpc*^−/−^ mutant mice at age embryonic day 18.5 (n = 4 and 5, respectively); heat map generated by RNA-seq analysis. *Qpc, Lhx1,* and wingless-type MMTV integration site family, member 4 *(Wnt4)* transcript levels were validated by quantitative real-time polymerase chain reaction (n = 3). (**c**) RNA fluorescent *in situ* hybridization analysis of *Wnt4* expression (white arrows) in kidney sections from *Cre*^−^ control littermates and *Six2-Qpc*^−/−^ kidneys at age P0. Ureteric buds are outlined by dashed white lines. Bar = 50 μm. Data are expressed as mean ± SEM; Student’s *t* test; 2-tailed, *P < 0.05, **P < 0.01. *18S,* 18S ribosomal RNA; CM, cap mesenchyme; CSB, comma-shaped bodies; *Fgf8,* fibroblast growth factor 8; *Lef1*, lymphoid enhancer-binding factor 1; Rel, relative; SSB, S-shaped bodies. To optimize viewing of this image, please see the online version of this article at www.kidney-international.org.

Phenotypic analysis of *Six2-Qpc*^−/−^ mutant kidneys suggested that mt electron transport chain disruption inhibited the ability of CM progenitors to differentiate. This notion was consistent with IF staining of *Six2-mT/mG;Qpc*^−/−^ kidneys for LIM homeobox protein 1 (LHX1), jagged 1 (JAG 1), and laminin, which indicated the absence of renal vesicle, comma-shaped body, and S-shaped body formation (Figure 4a).

To examine the role of *Qpc* in SIX2 progenitor cell differentiation on a transcriptional level, we took advantage of the fluorescent properties of Six2-Cre recombinase, which is fused to eGFP. We used fluorescence-activated cell sorting (FACS) to isolate *Six2-eGFP*/*cre*–expressing cells from heterozygous *Six2-Qpc*^+/−^ control and mutant *Six2-Qpc*^−/−^ kidneys at age E18.5.

We first assessed the degree of eGFP^+^ cell enrichment and examined the expression levels of *Qpc* and UB-associated transcripts in sorted cells. Transcript levels of UB-specific ret receptor tyrosine kinase and *Wnt9b* were not detectable in control and mutant eGFP^+^ cells, whereas *Qpc* expression was readily detectable in control but strongly reduced in mutant cells (relative mRNA levels of 0.04 ± 0.01 vs. 0.91 ± 0.25 in controls; n = 3 each, *P* = 0.004; Figure 4b and data not shown). We next performed genome-wide transcriptomic analysis of *eGFP*^+^ *Qpc*^−/−^ cells by RNA-seq analysis. RNA-seq analysis revealed that genes associated with MET and nascent nephron formation were downregulated in eGFP^+^ *Qpc*^−/−^ mutant compared with *eGFP*^+^ *Qpc*^+/−^ control cells, which was validated by quantitative real-time polymerase chain reaction. Downregulated genes included fibroblast growth factor 8 (*Fgf8*), lymphoid enhancer binding factor 1 (*Lef1*), wingless-type MMTV integration site family, member 4 (*Wnt4*), *Lhx1*, and *Jag1* (Figure 4b).^17–22^ Furthermore, disruption of renal vesicle formation in mutant kidneys was demonstrated by RNA fluorescent *in situ* hybridization for *Wnt4* (Figure 4c). Taken together, these data indicate that mt electron transport is necessary for differentiation of SIX2 progenitor cells into kidney tubules.

### SIX2 progenitor pool size is reduced in *Qpc*^−/−^ mutant mice

**Figure 5 |:**
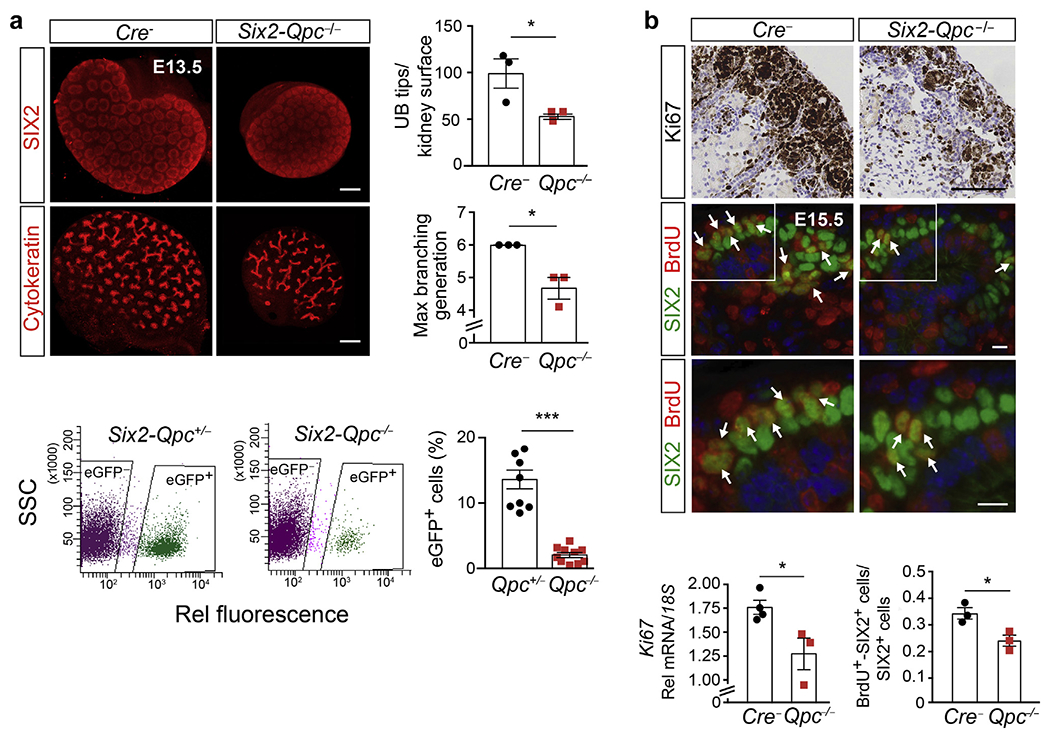
SIX2 progenitor proliferation is reduced in *Six2-Qpc*^−/−^ kidneys. (**a**) Representative immunofluorescence (IF) images of whole mounts from *Cre*^−^ littermate control and mutant *Six2-Qpc*^−/−^ kidneys at age embryonic day (E) 13.5, stained for SIX2 and cytokeratin. Shown on the right are ureteric bud (UB) tip numbers per kidney surface and maximal branching generations per kidney in control and mutant mice. Bar = 500 μm. Lower panels: quantification of *Six2-eGFP/cre*–expressing cells (eGFP^+^) inheterozygous control (*Six2-Qpc*^+/−^) and mutant *Six2-Qpc*^−/−^ kidneys at age E18.5. The number of eGFP^+^ cells is expressed as percentage of the total number of cells analyzed by fluorescence-activated cell sorting. Gating for *Six2-Qpc*^+/−^ and *Six2-Qpc*^−/−^ is shown on the right. The possibility of capturing differentiating *Qpc*^+/−^ cells with low GFP expression cannot be completely excluded. (**b**) Right upper panels: proliferative activity in *Six2-Qpc*^−/−^ kidneys, assessed by Ki67 immunohistochemistry (IHC) at age postnatal day 0 and bromodeoxyuridine (BrdU) labeling at age E15.5. The white arrows depict cells double positive for BrdU and SIX2. Right lower panels: *Ki67* transcript levels in whole kidney homogenates and ratio of BrdU/SIX2 double-positive cells (SIX2^+^BrdU^+^) to total number of SIX2^+^ cells in control and mutant kidneys. Bar = 100 μm for Ki67 IHC images, and Bar = 10 μm for BrdU IF images. Data are expressed as mean ± SEM; Student’s *t* test, 2-tailed, *P < 0.05 and ***P < 0.001. *18S*, 18S ribosomal RNA; Rel, relative. To optimize viewing of this image, please see the online version of this article at www.kidney-international.org.

Self-renewal of renal progenitors is crucial for nephrogenesis. Whole mount IF staining of embryonic kidneys at age E13.5 demonstrated that the number of SIX2^+^ caps per kidney, the number of ureteric tips, and the number of maximal UB branch generations were reduced in *Six2-Qpc*^−/−^ kidneys (Figure 5a). These findings are also reflected in a lower representation of eGFP^+^ cells in whole kidney cell isolates from *Six2-Qpc*^−/−^ mutants (2.06% ± 1.22% vs. 13.62% ± 4.33% in *Six2-Qpc*^+/−^ controls; n = 10 and 8, respectively, *P* < 0.001), suggesting that disrupted mt electron transport resulted in a reduction in the size of the SIX2 progenitor cell pool (Figure 5a).

To examine whether the reduction in SIX2 progenitor pool size was associated with decreased proliferation or increased apoptosis, we used immunohistochemistry to analyze the expression of proliferation marker Ki67 and apoptosis marker cleaved caspase 3. Furthermore, we performed bromodeoxyuridine (BrdU) labeling and terminal deoxynucleotidyl transferase–mediated dUTP nick end-labeling assays in *Six2-Qpc*^−/−^ and control kidneys at age E15.5 and P0, respectively. Ki67 immunohistochemistry at age P0 indicated that cell proliferation was reduced in the nephrogenic zone, which was also reflected in a decrease in *Ki67* transcript levels (Figure 5b). In line with these findings was the significant reduction in BrdU-labeled SIX2^+^ cells (BrdU^+^ SIX2^+^ to SIX2^+^ cell ratio of 0.24 ± 0.04 in *Six2-Qpc*^−/−^ kidneys vs. 0.34 ± 0.04 in *Cre*^−^ littermate controls; n = 3 each, *P* = 0.026). In contrast, immunohistochemistry analysis of cleaved-caspase 3 expression and terminal deoxynucleotidyl transferase–mediated dUTP nick end-labeling staining did not indicate significant differences between mutant and control kidneys (Supplementary Figure S5). Our data indicate that *Qpc* inactivation in SIX2 progenitors was associated with a reduction in proliferation and not with an increase in apoptosis.

Mitochondria are a source of ROS, which have been shown to cause DNA damage and suppress cellular proliferation.^23^ Because defects in mt electron transport impact on mt ROS production, we used CellROX staining to assess ROS levels in eGFP^+^ nephron progenitors isolated from mutant kidneys at age E18.5. Analysis by FACS did not identify a significant difference in mean fluorescence intensity between mutant and control mice, which is consistent with findings in other cell types (Supplementary Figure S6A).^4^ In addition, we stained kidney sections from newborn control and *Six2-Qpc*^−/−^ mice for 8-hydroxy-2′-deoxyguanosine, an oxidative DNA damage marker. Increased staining was not observed in mutant kidneys (Supplementary Figure S6B). Taken together, these data suggest that reduced proliferation in the *Qpc*^−/−^ CM was not due to increased ROS production.

### Growth factor-associated signaling pathways are activated in *Six2-Qpc*^−/−^ kidneys

To gain insight into the potential mechanisms underlying reduced progenitor proliferation in *Six2-Qpc*^−/−^ kidneys, we analyzed gene expression in SIX2 progenitors isolated by FACS at age E18.5. We found that multiple genes involved in the regulation of growth and proliferation, such as twist basic helix-loop-helix transcription factor 1 (*Twist1*) and myelomatosis oncogene c-M*yc*, were either equally expressed or upregulated compared with control (Supplementary Figure S7), suggesting that growth-associated signaling pathways were not suppressed in *Qpc*^−/−^ SIX2 progenitors.

**Figure 6 |:**
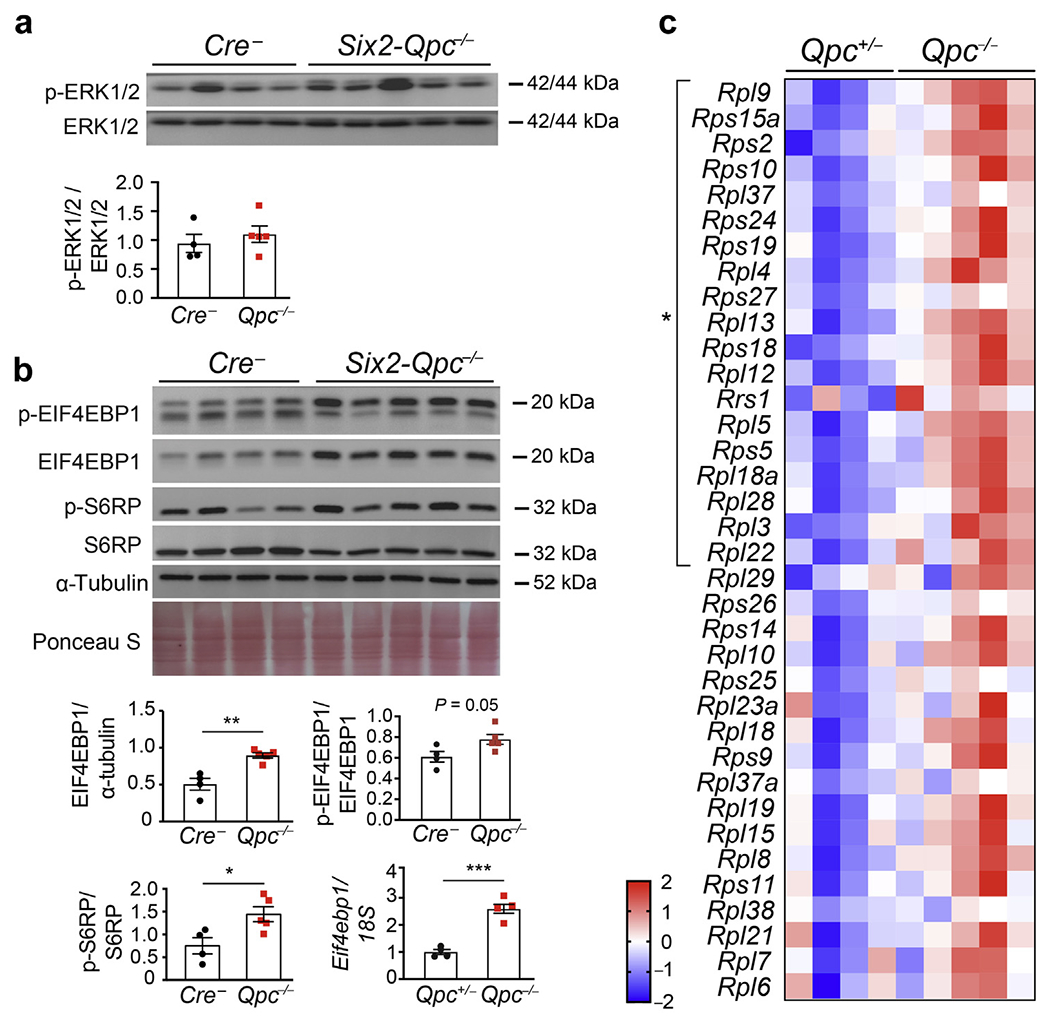
Growth factor–associated signaling pathways are activated in *Six2-Qpc*^−/−^ kidneys. (**a**) Phospho (p)-extracellular signal–regulated kinase (ERK)1/2 and total ERK1/2 protein levels in whole kidney lysates from *Cre*^−^ littermate control littermate and *Six2-Qpc*^−/−^ mutants at postnatal day (P) 0. Lower panel: ratio of p-ERK1/2 to ERK1/2 protein levels. (**b**) Immunoblot analysis of eukaryotic translation initiation factor 4E-binding protein 1 (EIF4EBP1), phospho (p)-EIF4EBP1, ribosomal protein S6 (S6RP), phospho (p)-S6RP, and α-tubulin in whole kidney lysates from *Cre*^−^ littermate control and *Six2-Qpc*^−/−^ mutant mice at age P0. Ponceau S staining to assess protein loading. Lower panels: EIF4EBP1/α-tubulin, p-EIF4EBP1/EIF4EBP1, and p-S6RP/S6RP ratios. Relative transcript levels of *Eif4ebp1* in *Six2-eGFP/cre–expressing* progenitor cells isolated from *Six2-Qpc*^+/−^ control and *Six2-Qpc*^−/−^ mice at age embryonic day (E) 18.5. (**c**) Relative expression levels of genes involved in ribosome biogenesis at E18.5; heat map generated by RNA-seq analysis of *Six2-eGFP/cre*–expressing progenitor cells isolated from heterozygous *Six2-Qpc*^+/−^ control and *Six2-Qpc*^−/−^ mutant mice (n = 4 and 5, respectively). Data are expressed as mean ± SEM; Student’s *t* test, 2-tailed, *P < 0.05, **P < 0.01, ***P < 0.001. *18S*, 18S ribosomal RNA.

We next investigated whether the activity of extracellular signal–regulated kinase (ERK)1/2, which is required for nephron progenitor maintenance and expansion,^24^ was abnormally regulated in *Six2-Qpc*^−/−^ kidneys. We found that protein levels of activated ERK1/2, phospho-ERK1/2, were not different from control kidneys (Figure 6a). Because of its crucial role in kidney development, we next investigated the mammalian target of rapamycin complex (mTORC) signaling pathway.^25^ We found increased phosphorylation of mTORC1 targets 40S ribosomal protein S6 (S6RP) and eukaryotic translation initiation factor 4E binding protein 1 (EIF4EBP1), indicating activation of mTORC1 signaling (Figure 6b). Furthermore, transcriptomic analysis of *Qpc*^−/−^ SIX2 progenitors revealed increased ribosomal gene expression (Figure 6c), which is consistent with mTORC1 activation.^26^ Taken together, our data suggest that despite the reduction in SIX2 progenitor proliferation, the activity of growth factor–associated signaling pathways involved in nephron progenitor maintenance and expansion was not decreased in *Six2-Qpc*^−/−^ kidneys.

### *Six2-Qpc*^−/−^ kidneys are characterized by a reduction in TCA cycle intermediates and amino acids

**Figure 7 |:**
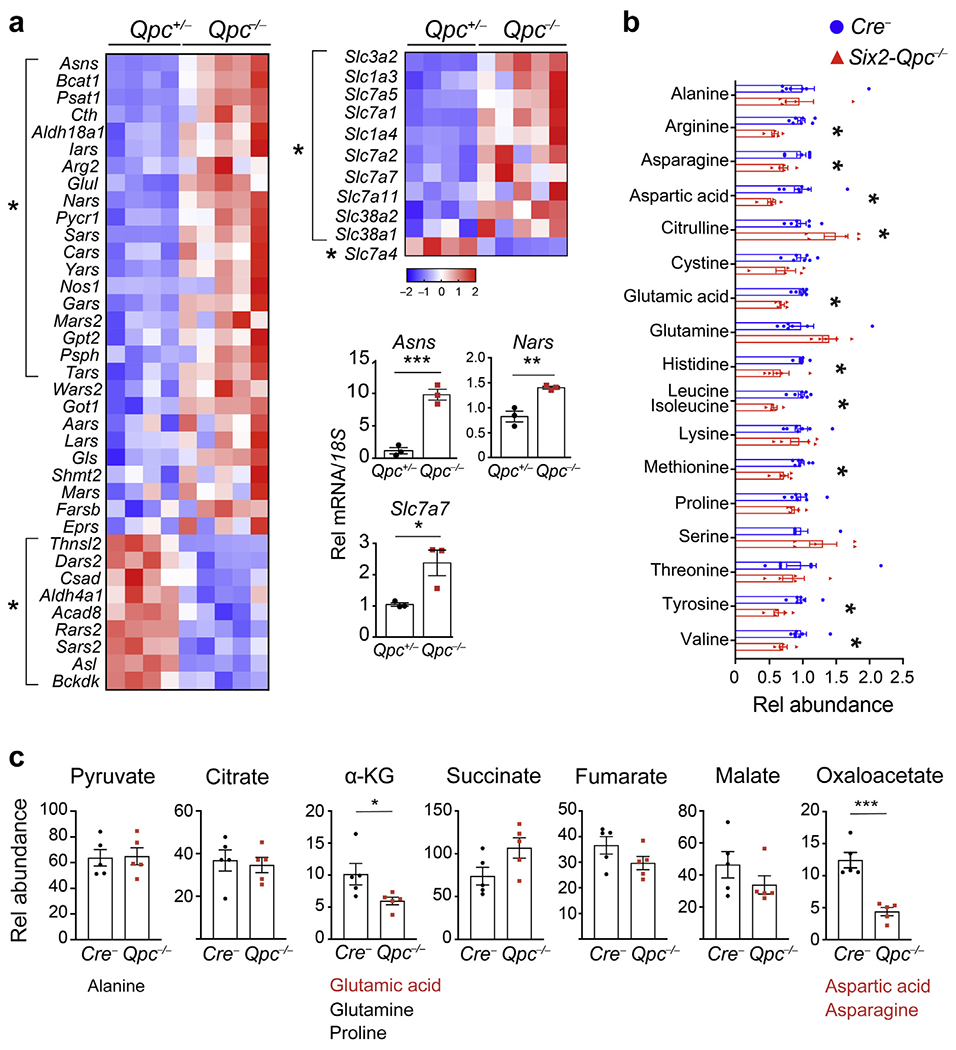
*Six2-Qpc*^−/−^ kidneys are characterized by amino acid deficiency. (**a**) Relative expression levels of genes involved in amino acid metabolism analyzed by RNA-seq at age embryonic day 18.5. *Six2-eGFP/cre*–expressing progenitor cells were isolated from heterozygous *Six2-Qpc*^+/−^ control and *Six2-Qpc*^−/−^ mice (n = 4 and 5, respectively). Lower-right panels: expression analysis of genes involved in asparagine synthesis, transport, and metabolism by quantitative real-time polymerase chain reaction; asparagine synthetase (*Asns*), solute carrier family 7 member 7 (*Slc7a7*, also known as y+L amino acid transporter 1), and nuclear encoded asparaginyl-tRNA synthetase (*Nars*). (**b**) Relative amino acid levels analyzed by mass spectrometry at age postnatal day 0; *Six2-Qpc*^−/−^ kidneys were compared with *Cre*^−^ littermate control kidneys (n = 5 and 7, respectively). (**c**) Relative levels of tricarboxylic acid cycle metabolites by mass spectrometry and metabolically associated nonessential aminoacids. Significantly decreased amino acids (n = 5) in *Six2-Qpc*^−/−^ kidneys are shown in red. Data are expressed as mean ± SEM; Student’s *t* test; 2-tailed,*P < 0.05, **P < 0.01, ***P < 0.001. *18S*, 18S ribosomal RNA; α-KG, α-ketoglutarate; Rel, relative.

Because amino acids are critical for cell growth, we investigated whether *Qpc* inactivation altered amino acid metabolism in the developing kidney. Transcriptomic analysis of *Qpc*^−/−^ SIX2 progenitors revealed that genes encoding amino acid transporters, amino acid synthetases, and aminoacyl-tRNA synthetases were differentially expressed; 29 genes were significantly upregulated, including asparagine synthetase (*Asns*), solute carrier family 7 member 7 (*Slc7a7*, also known as y+L amino acid transporter 1), and nuclear encoded asparaginyl-tRNA synthetase (*Nars*), and 10 were downregulated (Figure 7a).

We next used mass spectrometry to examine amino acid levels in whole kidney lysates from *Six2-Qpc*^−/−^ mutants. We found that tissue levels of 9 from 17 amino acid species analyzed were significantly decreased in *Six2-Qpc*^−/−^ kidneys compared with control (Figure 7b). These included the nonessential amino acids glutamic acid, aspartic acid, and asparagine, which are converted from TCA cycle intermediates α-ketoglutarate and oxaloacetate, but not serine and alanine, which are derived from glycolysis intermediates pyruvate and glyceraldehyde 3-phosphate.^27^ Aspartic acid and glutamic acid are directly involved in purine and pyrimidine synthesis and the cell’s ability to produce nucleotides.^28^ Tissue levels of aspartic acid and glutamic acid were reduced by approximately 50% and approximately 30%, respectively, oxaloacetate levels by approximately 65%, and α-ketoglutarate levels by approximately 40%, whereas pyruvate levels did not change. Purine and pyrimidine nucleotides adenosine, guanosine, inosine, cytidine, and uridine were reduced by approximately 25%–50% (Figure 7 and Supplementary Figure S8).

Amino acid deficiency induces activating transcription factor 4 (ATF4), a key effector of the integrated stress response to support protein synthesis for cell growth.^29,30^ In line with ATF4’s role in integrated stress response, amino acid metabolism, and nutrient sensing, we found increased transcript levels of ATF4-regulated stress response genes in *Qpc*^−/−^ SIX2 progenitor cells, which included *Asns*, *Slc7a7*, *Nars*, ATF3 (*Atf3*), tribbles pseudokinase 3 (*Trib3*), and DNA damage inducible transcript 3 (*Ddit3*; Figure 7 and Supplementary Figure S9).^31–34^ Taken together, our findings suggest that *Six2-Qpc*^−/−^ kidneys were characterized by depletion of TCA cycle intermediates and amino acid deficiency, which in turn resulted in reduced purine and pyrimidine synthesis and activation of ATF4-mediated stress responses.

## DISCUSSION

Here we investigated that the role of mt electron transport in kidney development and homeostasis by genetically inactivating mt complex III subunit QPC. We found that disruption of mt electron transport in SIX2 progenitors but not in UB epithelium resulted in severely dysplastic kidneys due to the failure of SIX2 progenitors to expand and undergo MET. Decreased proliferation of *Qpc*^−/−^ SIX2 progenitor cells was associated with a reduction in kidney TCA cycle metabolites limiting amino acid and nucleotide synthesis.

Although the effects of *Qpc* inactivation in SIX2 progenitors on kidney development are dramatic, the effects of homozygous *Qpc* inactivation in the adult kidney are less clear and will have to be studied experimentally with genetically inducible models. Common kidney manifestations of mt electron transport defects in patients include tubulointerstitial injury and isolated tubular dysfunction, for example, Fanconi’s syndrome, or renal tubular acidosis.^35–37^ Nephrotic syndrome is less common and frequently involves defects in coenzyme Q10 synthesis.^36^ Primary mt complex III deficiencies, such as mutations in mitochondrially encoded cytochrome b, mt complex III assembly protein BCSL1 (BCS1 homolog, ubiquinol-cytochrome c reductase complex chaperone), or QPC, are rare. They can affect multiple organ systems with varying complexity and severity, for example, hepatic failure and encephalopathy associated with tubulointerstitial disease in patients with BCSL1 mutations, and may result in infantile lethality.^38–43^ Homozygosity for a nonlethal, autosomal recessive missense mutation in QPC has been described in 25 related individuals with severe psychomotor retardation and lactic acidosis; however, a kidney phenotype was not reported in these patients.^40^ Whereas the complete loss of QPC function in humans is likely to result in early embryonic lethality, it is unclear whether QPC haploinsufficiency predisposes to or causes disease. Although we have not observed a spontaneous kidney disease phenotype in mice that lack 1 copy of *Qpc* in SIX2-derived kidney epithelial cells, it plausible that *Six2-Qpc*^+/−^ heterozygotes might be more susceptible to kidney injury. This possibility is currently under investigation in our laboratory.

Defective nephrogenesis in *Six2-Qpc*^−/−^ mutants is likely to result from multiple mechanisms including the metabolic consequences of TCA cycle suppression and possibly the reduced oxidative phosphorylation–dependent production of mt ATP. Studies in pluripotent stem cells have shown that the initiation of cellular differentiation is associated with a switch from glycolysis to mt oxidative metabolism.^44^ These observations are in line with our findings and the observation that inhibition of glycolysis in SIX2 progenitor cells promoted their differentiation, resulting in SIX2 progenitor depletion.^45^ In contrast, an increase in glycolytic flux generated by hypoxia-inducible factor activation resulted in an expansion of the SIX2 progenitor cell pool and inhibited nephron progenitor cell differentiation, which was associated with decreased nephron numbers.^46^ Increased glycolysis is a metabolic hallmark of proliferating cells and not only supports anaerobic ATP generation but also shunts intermediary metabolites into anabolic pathways, such as the pentose phosphate pathway.^44,47^

Our data suggest that mt ATP generation is not required for UB differentiation and development of the kidney collecting system, which is metabolically different from the proximal nephron. Mitochondrial density, as assessed by the distribution of mitochondrial enzymes along the nephron, is low in collecting tubules and high in the proximal tubule and loop of Henle.^48^ Furthermore, collecting duct segments are highly glycolytic and characterized by increased lactate production, particularly in the inner medulla, which is known to be hypoxic with tissue pO_2_ levels of <10 mm Hg.^49,50^ It is therefore conceivable that the development of the kidney collecting system is less susceptible to disruptions of mt oxidative metabolism due to its baseline glycolytic profile. To what degree *Qpc* inactivation in UB epithelial cells suppresses TCA cycle flux is not clear from our study. Because TCA cycle flux is dependent on the availability NAD^+^, the degree of TCA cycle suppression in *Qpc*-deficient cells will depend on the degree of mt NAD^+^ depletion. It is possible that UB epithelial cells generate adequate amounts of NAD^+^ from sources other than mt electron transport, permitting TCA cycle reactions to occur at sufficient rates. NAD^+^/NADH ratios, TCA cycle metabolite concentrations, and glycolytic flux measurements would have to be studied in UB cells specifically to test these hypotheses. Although the formation of the kidney collecting system was not inhibited, kidneys from *Hoxb7-Qpc*^−/−^ mice were smaller. Therefore, we cannot exclude that mt electron transport chain disruption had effects on UB epithelial cell proliferation. Alternatively, complex III deficiency in UB progenitors may have impacted on UB-CM reciprocal interactions and signaling, the degree of which is unclear and warrants further investigation. However, despite smaller kidneys, blood urea nitrogen levels in adult *Hoxb7-Qpc*^−/−^ mice were within normal limits.

Mitochondria are complex metabolic signaling organelles where electron transport intersects with multiple pathways, including TCA cycle, amino acid, fatty acid, glucose, and 1 carbon metabolism.^1–3^ Beyond the generation of ATP, dysregulated mt electron transport impacts on ROS production, cellular redox state, that is, the NAD^+^/NADH ratio, apoptosis, proliferation, and cellular differentiation. Several studies have demonstrated that inhibition of complex I or III reduces cellular proliferation.^51–54^ One of the proposed mechanisms by which this occurs is the reduction in aspartate synthesis, which is critical for the generation of nucleotides and is constrained by low NAD^+^ levels.^55,56^ In line with these observations are our findings of decreased oxaloacetate and aspartate levels in *Six2-Qpc*^−/−^ kidneys, which is likely to have contributed to the reduced proliferation of *Qpc*^−/−^ SIX2 progenitors. In addition to aspartate, *Six2-Qpc*^−/−^ kidneys were characterized by reductions in glutamic acid, asparagine, arginine, and the essential amino acids valine, leucine, isoleucine, histidine, methionine, and threonine. Consistent with our findings in *Six2-Qpc*^−/−^ kidneys, depletion of both essential and nonessential amino acid was found in *Qpc*-deficient lung endothelial cells.^54^ A reduction in cerebral amino acids was also described in mice with genetic inactivation of mt complex I subunit NDUFS4, which develop clinical manifestations of Leigh syndrome, a pediatric neurological disorder characterized by progressive psychomotor regression.^57^ However, we recognize that differences in cellular composition between control and mutant kidneys may limit the interpretation of our metabolic analysis by mass spectrometry.

*Six2-Qpc*^−/−^ kidneys were characterized by a relative increase in mTORC1 activity compared with control. mTOR functions as a serine/threonine protein kinase that integrates input from upstream growth factor receptors with cellular ATP and amino acid availability. mTOR activation promotes protein synthesis, cell proliferation, and survival, whereas conditions of energy stress or limited nutrient availability inhibit mTOR signaling.^58,59^ Although counterintuitive, mTOR activity was not downregulated but increased in *Six2-Qpc*^−/−^ kidneys. We suggest that this could be the result of continued growth factor stimulation of CM progenitors, indicating that mTOR regulation is uncoupled from amino acid sensing in*Six2-Qpc*^−/−^ kidneys.

**Figure 8 |:**
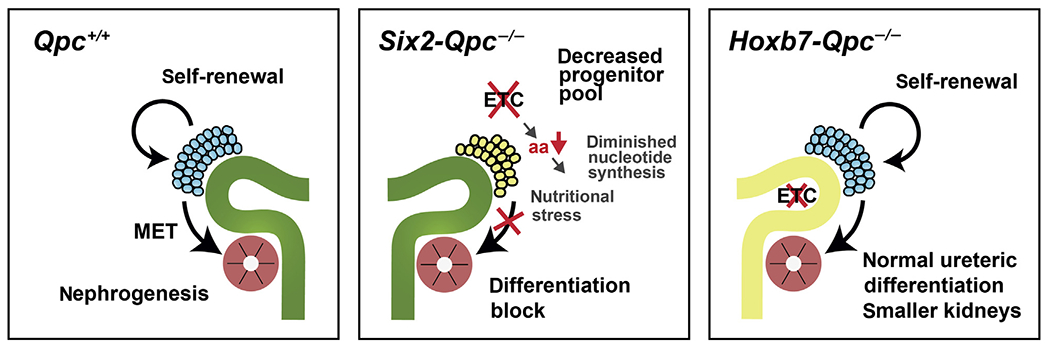
Schematic depicting the consequences of mitochondrial complex III disruption in kidney progenitors. Normal nephron development is governed by a delicate balance between nephron progenitor self-renewal and differentiation (left panel). Disruption of mitochondrial complex III in SIX2 progenitor cells is associated with amino acid (aa) depletion, reduced nucleotide synthesis, and inhibition of cap mesenchyme progenitor proliferation and differentiation, whereas disruption of mitochondrial complex III in HOXB7 ureteric progenitors does not block the development of the kidney’s collecting system. ETC, mitochondrial electron transport chain; MET, mesenchymal to epithelial transition.

In summary, our studies establish a differential role for mt electron transport in the proliferation and differentiation of kidney progenitor cells. Our data identify critical links between mt electron transport, TCA cycle, amino acid, and nucleotide synthesis that regulate CM progenitor maintenance and differentiation (Figure 8). Pharmacologic targeting of metabolic pathways may therefore be useful for therapeutic manipulations of nephrogenesis to enhance nephron endowment.

## Supplementary Material

Guan_et_al_Supp_Mat Supplementary Methods.
**Figure S1.** Nephron formation is defective in *Six2-Qpc*^−/−^ mutant mice (associated with Figures 1 and 2).
**Figure S2.** Nephron segment–specific gene expression at age embryonic day (E) 15.5 (associated with Figure 2).
**Figure S3.** *Qpc* inactivation in the ureteric bud trunk and tip (associated with Figure 3).
**Figure S4.** Analysis of *Hoxb7-Qpc*^−−^ mutants at age postnatal day (P) 0 (associated with Figure 3).
**Figure S5.** Apoptosis is not increased in *Six2-Qpc*^−/−^ mutants (associated with Figure 5).
**Figure S6.** *Qpc* inactivation in SIX2 progenitors does not increase reactive oxygen species (ROS) levels (associated with Figure 5).
**Figure S7.** Growth factor–activated signaling pathways are not downregulated in *Six2-Qpc*^−/−^ kidneys (associated with Figure 6).
**Figure S8.** Purine and pyrimidine metabolites are reduced in *Six2-Qpc*^−/−^ kidneys (associated with Figure 7).
**Figure S9.** Activation of activating transcription factor 4 (ATF4)–mediated stress responses in *Six2-Qpc*^−/−^ kidneys (associated with Figure 7).
**Table S1.** Primer sequences.
**Table S2.** Primary antibodies used for immunohistochemistry, immunofluorescence, or immunoblotting.
Supplementary References.

## Translational Statement

Mitochondria support energy homeostasis in the kidney, regulate cellular differentiation, and play a critical role in kidney pathogenesis. This study used a genetic approach to investigate mitochondrial electron transport in nephron development and kidney homeostasis. Unexpectedly, mitochondrial electron transport was dispensable for the development and maintenance of the kidney collecting duct system. In contrast, disruption of mitochondrial electron transport in cap mesenchyme inhibited nephron formation, highlighting compartment-specific needs for metabolic reprogramming during kidney development. Our studies identify a critical link between defective mitochondrial electron transport and amino acid metabolism impacting nephron differentiation and growth.
