# Supplementary material for: Disruption of mitochondrial complex III in cap mesenchyme but not in ureteric progenitors results in defective nephrogenesis associated with amino acid deficiency
Source: Kidney Int. Author manuscript; Available in PMC 2022 Jul 1. (PMC9232975; doi:10.1016/j.kint.2022.02.030)
Supplement: Guan_et_al_Supp_Mat — Supplementary Methods. Figure S1. Nephron formation is defective in Six2-Qpc−/− mutant mice (associated with Figures 1 and 2). Figure S2. Nephron segment–specific gene expression at age embryonic day (E) 15.5 (associated with Figure 2). Figure S3. Qpc inactivation in the ureteric bud trunk and tip (associated with Figure 3). Figure S4. Analysis of Hoxb7-Qpc−− mutants at age postnatal day (P) 0 (associated with Figure 3). Figure S5. Apoptosis is not increased in Six2-Qpc−/− mutants (associated with Figure 5). Figure S6. Qpc inactivation in SIX2 progenitors does not increase reactive oxygen species (ROS) levels (associated with Figure 5). Figure S7. Growth factor–activated signaling pathways are not downregulated in Six2-Qpc−/− kidneys (associated with Figure 6). Figure S8. Purine and pyrimidine metabolites are reduced in Six2-Qpc−/− kidneys (associated with Figure 7). Figure S9. Activation of activating transcription factor 4 (ATF4)–mediated stress responses in Six2-Qpc−/− kidneys (associated with Figure 7). Table S1. Primer sequences. Table S2. Primary antibodies used for immunohistochemistry, immunofluorescence, or immunoblotting. Supplementary References. [file NIHMS1794010-supplement-Guan_et_al_Supp_Mat.pdf]

SUPPLEMENTARY MATERIALS

Nan Guan, Hanako Kobayashi, Ken Ishii, Olena Davidoff, Feng Sha, Talat A. Ikizler, Chuan-Ming Hao, Navdeep S. Chandel and Volker H. Haase

Table of Contents:

1. Materials and Methods
2. Supplementary Tables
3. Supplementary Figures
4. References

3. MATERIALS and METHODS

DNA and RNA analysis. Genomic DNA analysis was performed as previously described.[1] RNA was isolated using the RNeasy kit according to the manufacturer's protocol (Qiagen; Hilden, Germany). Quantitative real-time PCR (qPCR) was performed as described previously.[2] *18S* rRNA was used for normalization. For the quantification of mRNA expression, the relative standard curve method was used; separate standard curves were generated for each qPCR run. Primer sequences used for the detection of *Aqp1*, *Aqp2*, *Lhx1*, nephrin, podocin, *Napi2a*, *Ncc*, *Nkcc2*, *Scnn1a*, *Six2* and *Trpv5* transcripts have been described previously,[3] otherwise primer sequences are listed in Supplementary Table S1.

For RNA FISH, *Wnt4* or *Qpc* were detected in formalin-fixed, paraffin-embedded kidney tissue sections from *Six2-Qpc*$^{-/-}$ and *Hoxb7-Qpc*$^{-/-}$ mice, respectively, using the RNAscope® Multiplex Fluorescent kit or Basescope™ Colormetric kit according to the manufacturer's instructions (Advanced Cell Diagnostics; Newark, USA). Tissue slides were imaged on an Apiro Versa 200 automated slide scanner (Leica Biosystems; Wetzlar, Germany). RNA sequencing analysis was performed at age E18.5 with eGFP^{+} cells isolated by FACS from *Six2-Qpc*$^{+/-}$ control and *Six2-Qpc*$^{-/-}$ embryonic kidneys (Novogene; Beijing, China). For the generation of heat maps the mean FPKM was subtracted from the sample FPKM and divided by the sample FPKM standard deviation.

Morphologic analysis. Kidneys were fixed in 10% formalin and then embedded in paraffin. For morphologic evaluation, kidney sections were stained with H&E and toluidine blue. Antibodies used for IHC and IF analyses are listed in Supplementary Table S2. For lectin-histochemistry, sections were incubated with lotus tetragonolobus lectin, LTL (Vector Laboratories; Burlingame, CA, USA; catalog # FL-1321) or dolichos biflorus agglutinin, DBA (Vector Laboratories; Burlingame, CA, USA; catalog # B-1035). Slides were scanned with a Leica DMRXA2 3D/de-convolution scope or a Leica SCN400 slide scanner (Leica Biosystems; Wetzlar, Germany). Whole mount staining was performed with E13.5 embryonic kidneys as previously described and imaged using confocal microscopy.[4] For BrdU labeling, pregnant mice were injected i.p. with BrdU at a dose of 0.1 mg/g body weight (Abcam;

Burlingame, CA, USA; catalog # ab142567). Embryos were collected 6 hours after BrdU injection. Slides were scanned with a Leica DMRXA2 3D/de-convolution scope or a Leica SCN400 slide scanner (Leica Biosystems; Wetzlar, Germany).

Protein analysis. Total kidney protein lysates (40 µg) were analyzed by SDS-PAGE (Novex®, Invitrogen; Carlsbad, CA, USA) and transferred to nitrocellulose membranes (Novex®, Invitrogen; Carlsbad, CA, USA). Primary antibodies used for immunoblotting are listed in Supplementary Table S2. Ponceau S staining was performed to assess sample loading.

Metabolite analysis. For amino acid and nucleotide analysis, snap-frozen kidneys from *Cre*$^{-}$ littermate control and *Six2-Qpc*$^{-/-}$ pups at age P0 were pooled and homogenized in cold water/methanol (9:1) containing 50 mM ammonium acetate (pH 6). A portion of the homogenate (100 µL) was combined with HPLC-grade methanol (300 µL) vortexed and centrifuged at 10,000 x *g* for 10 min at 4 °C to remove precipitated proteins and other insoluble debris. The supernatant was transferred to a clean tube, dried and reconstituted in 100 µl of acetonitrile/water (2:1) containing 250 µM tyrosine (phenyl-3,5-d_2) internal standard. Amino acid species were measured by a targeted HILIC-MS/MS method. Individual reference standards of all analytes were infused into the mass spectrometer for the optimization of electrospray ionization (ESI) and selected reaction monitoring (SRM) parameters. LC-MS analysis was performed using an Acquity UPLC system (Waters; Milford, MA) interfaced with a TSQ Quantum Ultra triple-stage quadrupole mass spectrometer (Thermo Scientific, San Jose, CA). The mass spectrometer was equipped with an IonMax source housing and a heated ESI probe. Detection was based on SRM using the following optimized source parameters (positive ionization): spray voltage 5 kV; capillary temperature 300°C; vaporizer temperature 185 °C, tube lens 52 V at *m/z* 184; N_2 sheath gas 50; N_2 auxiliary gas 5. Data acquisition and quantitative spectral analysis were done using Thermo-Finnigan Xcalibur version 2.0.7 SP1 and Thermo-Finnigan LCQuan version 2.5.6, respectively. A SeQuant® ZIC®-cHILIC analytical column (3 µm, 100Å), PEEK 150 x 2.1 mm was used for all chromatographic separations (Merck KGaA; Darmstadt, Germany). Mobile

phases were made up of 0.2% acetic acid and 15 mM ammonium acetate in (A) H_2O/CH_3CN (9:1) and in (B) $CH_3CN/CH_3OH/H_2O$ (90:5:5). Gradient conditions were as follows: 0–2 min, B = 85%; 2–5 min, B = 85–30%; 5–9 min, B = 30%; 9–11 min, B = 30–85%; 11–20 min, B = 85%. The flow rate was maintained at 300 µL/min; the total chromatographic run time was 20 min. The sample injection volume was 10 µL. The autosampler injection valve and the sample injection needle were flushed and washed sequentially with mobile phase A (1 mL) and mobile phase B (1 mL) between each injection.

GC-MS analyses of TCA cycle metabolites were performed with an Agilent7890A gas chromatograph (Agilent Technologies; Wilmington, DE, USA) coupled with an Agilent 5975C mass-selective detector (MSD) as previously described.[5]

Blood urea nitrogen levels were analyzed by the Translational Pathology Shared Resource core at Vanderbilt University Medical Center. NAD^+/NADH ratios in kidneys were measured with the NAD/NADH Assay Kit according to the manufacturer's protocol (Abcam; Burlingame, CA, USA; catalog # ab65348).

TUNEL assay and ROS analysis. Formalin-fixed and paraffin-embedded kidney sections were pretreated and labeled with the DeadEnd™ Fluorometric TUNEL System (Promega Biosciences; San Luis Obispo, USA; catalog # G3250) following the manufacturer's instruction. For ROS analysis, kidneys were dissected from heterozygous Six2-Qpc$^{+/-}$ control and Six2-Qpc$^{-/-}$ embryos at age E18.5 and digested into single cell suspension. Samples were incubated with CellROX® (Invitrogen; Waltham, Massachusetts, USA; catalog # C10422) following the manufacturer's instruction and analyzed by flow cytometry.

2. SUPPLEMENTARY TABLES

Table S1: Primer sequences

Gene Name	Sense	Anti-Sense
Asns	GCAGTGTCTGAGTGCGATGAA	TCTTATCGGCTGCATTCCAAAC
Atf4	ATGGCGCTCTTCACGAAATC	ACTGGTCGAAGGGGTCATCAA
Atp6v1b1	CTGTGACCCGAAACTACATCAC	AAGTCCCTTCAAACACCTGAAC
Cited1	CCACTAGCTCCTCTGGATCG	AGCCCCTTGGTACTGGCTAT
Ki67	CAGTACTCGGAATGCAGCAA	CAGTCTTCAGGGGCTCTGTC
Nars	GAGCTGTATGTATCTGACCGAGA	AAATGGTGGGAAATGGCTCTTT
Qpc	CGCGTCTATCTTCTGTCCCA	CTGCTCAAACTCCTGGTTGC
Slc7a7	CATAGTGGGGAACATGATCGG	AGCCCAGATGACCAGTGAGA
Trib3	GCAAAGCGGCTGATGTCTG	AGAGTCGTGGAATGGGTATCTG
Wnt4	CGAGCAATTGGCTGTACCTG	TCCGGAACTGGTATTGGCAC

Table S2: Primary antibodies used for IHC, IF or immunoblotting

Primary antibody	Company	Catalog #
ATP6V1B1	Santa Cruz Biotechnology, Dallas, TX, USA	sc271832
AQP2	Sigma-Aldrich, St. Louis, MO, USA	SAB5200110
BrdU	Santa Cruz Biotechnology, Dallas, TX, USA	sc32323
c-caspase 3	Cell Signaling Technology, Danvers, MA, USA	9664
eGFP	Abcam, Burlingame, CA, USA	ab13970
EIF4EBP1	Cell Signaling Technology, Danvers, MA, USA	9644
ERK1/2	Cell Signaling Technology, Danvers, MA, USA	4695
JAG1	Developmental Studies Hybridoma Bank, Iowa City, IA, USA	TS1.15H
Ki67	Cell Signaling Technology, Danvers, MA	12202S
laminin	Sigma Aldrich, St. Louis, MO, USA	AB2034
LHX1	Developmental Studies Hybridoma Bank, Iowa City, IA, USA	4F2
megalin	Abcam, Burlingame, CA, USA	ab76969
NCC	StressMarq, Victoria, Canada	SPC-402
pan-cytokeratin	Sigma Aldrich, St. Louis, MO, USA	C2562
phospho-EIF4EBP1 (Thr37/46)	Cell Signaling Technology, Danvers, MA, USA	2855
phospho-Erk1/2 (Thr202/Tyr204)	Cell Signaling Technology, Danvers, MA, USA	4370
phospho-S6RP (Ser235/236)	Cell Signaling Technology, Danvers, MA, USA	4858
SIX2	Proteintech, Rosemont, Il, USA	11562-1-AP
S6RP	Cell Signaling Technology, Danvers, MA, USA	2217
α-tubulin	Cell Signaling Technology, Danvers, MA, USA	3873
WT1	Abcam, Burlingame, CA, USA	ab15249
8-OHdG	Adipogen, San Diego, CA, USA	501015749

3. SUPPLEMENTARY FIGURES

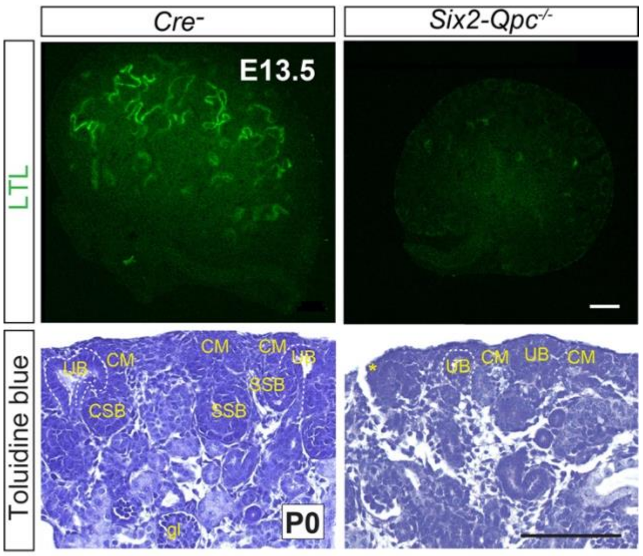

Supplementary Figure S1 (associated with Figures 1 and 2). Nephron formation is defective in *Six2-Qpc*$^{-/-}$ mutant mice. Upper panels, representative images of lotus tetragonolobus lectin (LTL) histochemistry of whole mount kidneys from *Cre*$^{-}$ control and *Six2-Qpc*$^{-/-}$ mice at age E13.5. Scale bar, 200 µm. Lower panels, toluidine staining of formalin-fixed, paraffin-embedded sections from *Cre*$^{-}$ control and *Six2-Qpc*$^{-/-}$ kidneys at age P0. Scale bar, 100 µm.

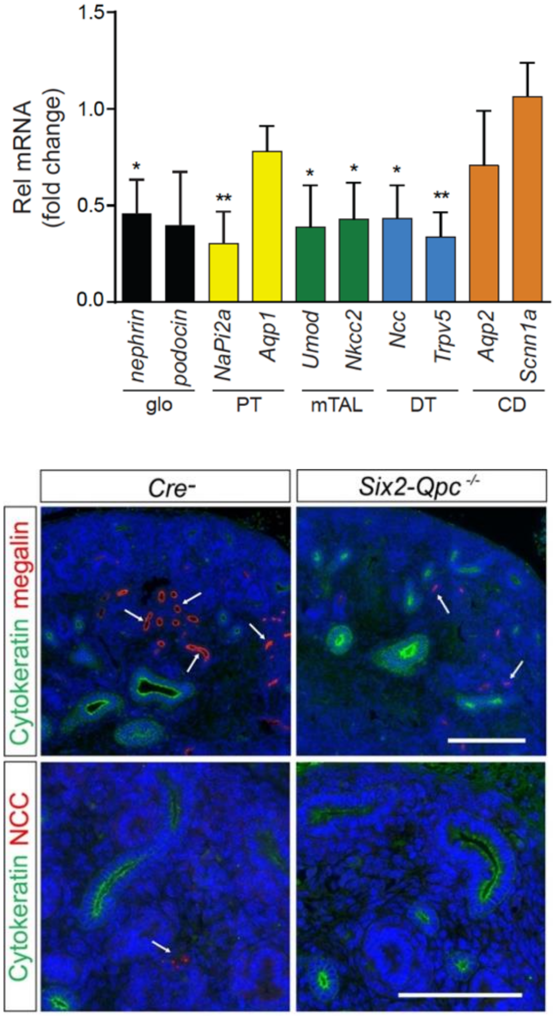

Supplementary Figure S2 (associated with Figure 2). Nephron segment-specific gene expression at age embryonic day (E) 15.5. Upper panel: Nephron segment-specific gene expression at age E15.5. Transcript levels were quantified in total RNA isolated from whole kidney extracts and compared with *Cre*$^{-}$ littermate controls; n=4 litters, except for *Umod* and *Aqp2* (n=3 litters). Data are presented as mean ± SEM; Student's *t*-test, two-tailed, *$P<0.05$, and **$P<0.0$. Lower panels: Immunofluorescence staining of differentiated nephron segment markers megalin and NCC at age E15.5. White arrows indicate positive staining. Scale bars, 200µm for megalin and 100 µm for NCC. Abb.: *Aqp1*, aquaporin 1; *Aqp2*, aquaporin 2; CD, collecting duct; DT, distal tubule; glo, glomerulus; mTAL, medullary thick ascending loop of Henle; *Ncc*, thiazide-sensitive sodium chloride cotransporter; *NaPi2a*, sodium-phosphate cotransporter 2A; *Nkcc2*, sodium-potassium-chloride cotransporter 2; PT, proximal tubule; Rel, relative; *Scnn1a*, epithelial sodium channel 1 alpha subunit; *Trpv5*, transient receptor potential cation channel subfamily V member 5; *Umod*, uromodulin.

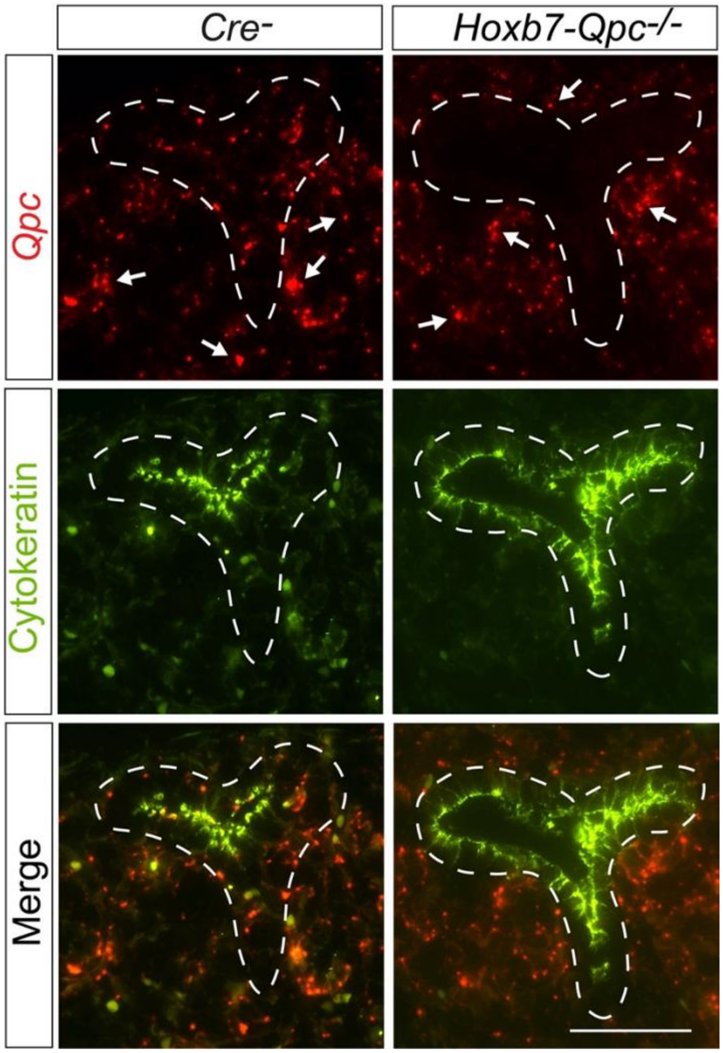

Supplementary Figure S3 (associated with Figure 3). *Qpc* inactivation in ureteric bud trunk and tip. Representative images of RNA fluorescent in situ hybridization (Basescope™), detecting the presence or absence of the targeted *Qpc* exon 1 sequence. Ureteric bud was visualized in embryonic kidney sections by simultaneous immunofluorescence for ureteric bud (UB) marker cytokeratin in *Cre*$^{-}$ littermate control and *Hoxb7-Qpc*$^{-/-}$ mutants at age E16.5. Arrows depict red fluorescent signals indicating the presence of the targeted *Qpc* exon 1 sequence. Scale bar, 50 µm.

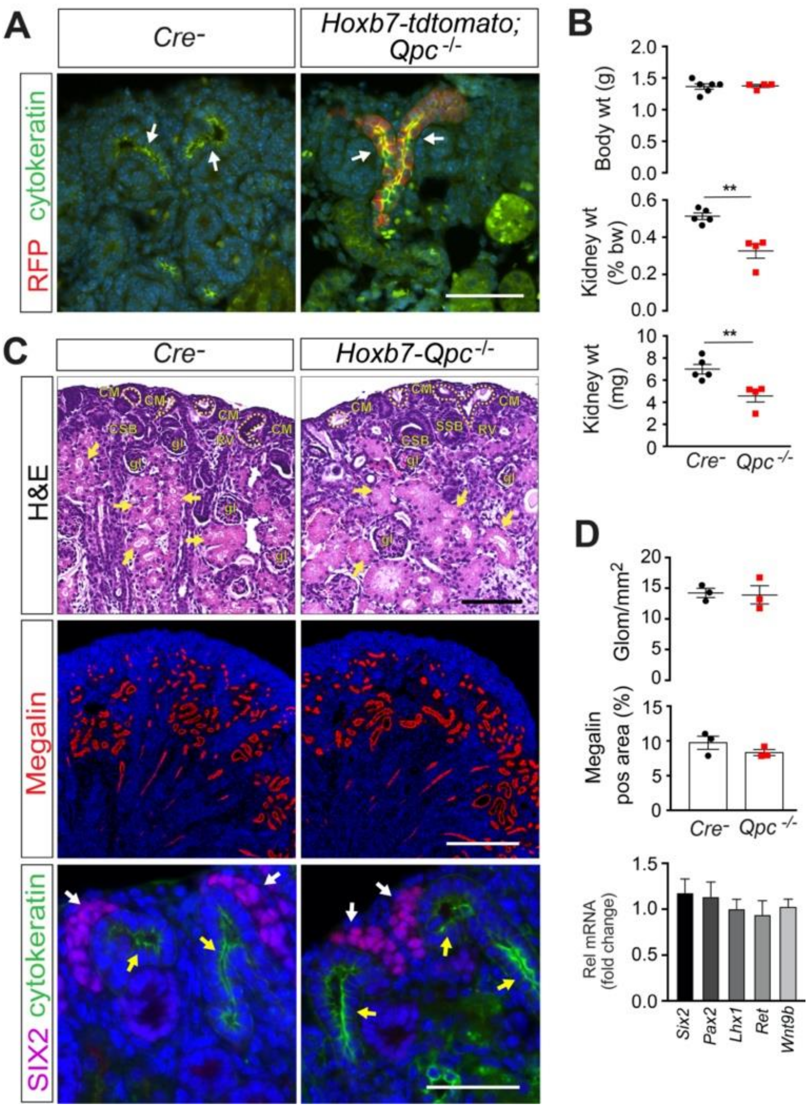

Supplementary Figure S4 (associated with Figure 3). Analysis of *Hoxb7-Qpc*$^{-/-}$ mutants at age postnatal day (P) 0. A) Shown are representative immunofluorescence (IF) images of kidney sections from *Cre*$^{-}$ littermate control and mutant *Hoxb7-tdtomato*;*Qpc*$^{-/-}$ mice at age P0, stained for red fluorescent protein (RFP) and cytokeratin. The presence of RFP indicates recombination of the Cre reporter allele (*tdtomato*). Arrows depict a ureteric bud (UB). Scale bar, 50 µm. (B) Total body weight, kidney weight and kidney/body weight ratio for control and *Hoxb7-Qpc*$^{-/-}$ mutants at age P0 (n=5 and 4, respectively). (C) Shown are representative images of kidney sections from control and *Hoxb7-Qpc*$^{-/-}$ mutants at age P0 stained with H&E and IF for megalin, Six2, and cytokeratin. Upper panels (H&E), UBs are outlined by dashed lines, yellow arrows indicate differentiated kidney tubules. Scale bar, 100 µm. Mid panels (megalin), scale bar, 300µm. Lower panels (SIX2/cytokeratin), white arrows depict cap mesenchyme, and yellow arrows depict UBs. Scale bar, 50µm. (D) Quantification of glomerular number and megalin expressing nephron segments per tissue area (n=3). Transcript levels of genes associated with mesenchymal to epithelial transition and nascent nephron formation in total kidney homogenates from control and *Hoxb7-Qpc*$^{-/-}$ mutants at age P0 (n=5). Data are presented as mean ± SEM; Student's *t*-test, 2-tailed, $^{**}P<0.01$. Abb.: CM, cap mesenchyme; CSB, comma-shaped body; gl, glomerulus; *Lhx1*, LIM homeobox 1; *Pax2*; paired box gene 2, Rel, relative; *Ret*; ret proto-oncogene; RV, renal vesicle; SSB; S-shaped body; *Wnt9b*, wingless-type MMTV integration site family, member 9b.

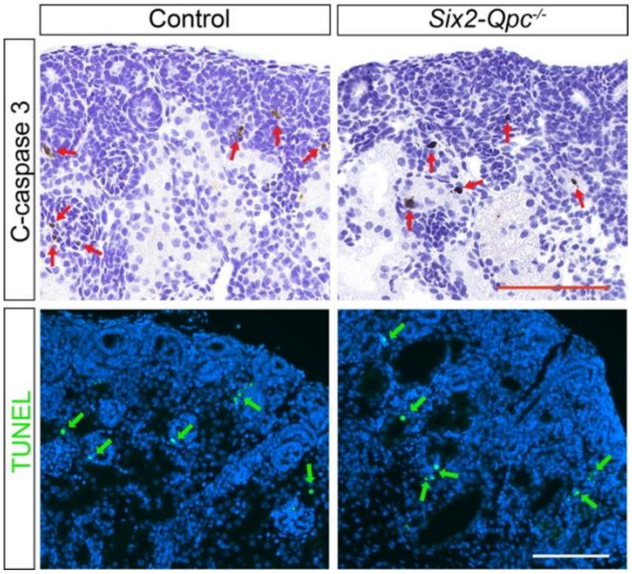

Supplementary Figure S5 (associated with Figure 5). Apoptosis is not increased in *Six2-Qpc$^{-/-}$* mutants. Representative images of kidneys sections from *Cre^{-}* littermate control and *Six2-Qpc$^{-/-}$* mice at age P0 stained for cleaved caspase 3 and analyzed by terminal deoxynucleotidyl transferase dUTP nick end labeling (TUNEL) assay. Scale bars, 100 µm.

Supplementary Figure S6 (associated with Figure 5). *Qpc* inactivation in SIX2 progenitors does not increase reactive oxygen species (ROS) levels. (A) Cellular ROS levels in SIX2 nephron progenitors assessed by flow cytometry; SIX2 progenitors were isolated from heterozygous *Six2-Qpc$^{+/-}$* control and *Six2-Qpc$^{-/-}$* kidneys at age E18.5. Shown are mean fluorescent intensities (MFI). (B) Representative images of kidney sections from *Cre^{-}* littermate control and *Six2-Qpc$^{-/-}$* mutants at age P0. Sections were stained for oxidative DNA damage marker 8-hydroxy-2'-deoxyguanosine (8OHdG) and SIX2 (green fluorescence, white arrows). Scale bar, 50 µm. Data are presented as mean ± SEM; Student's *t*-test, 2-tailed.

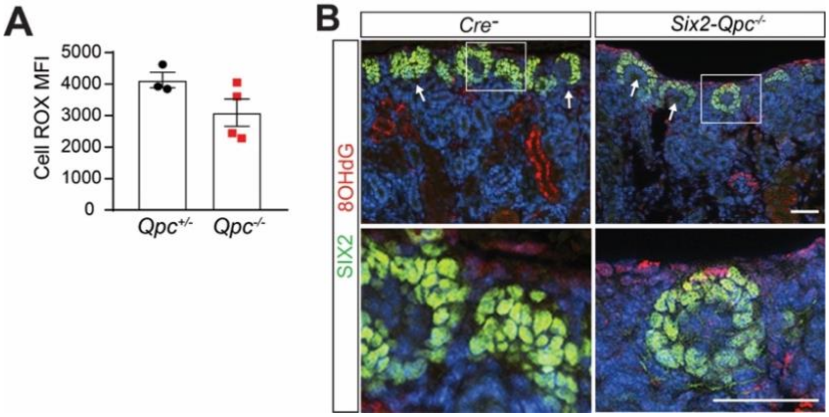

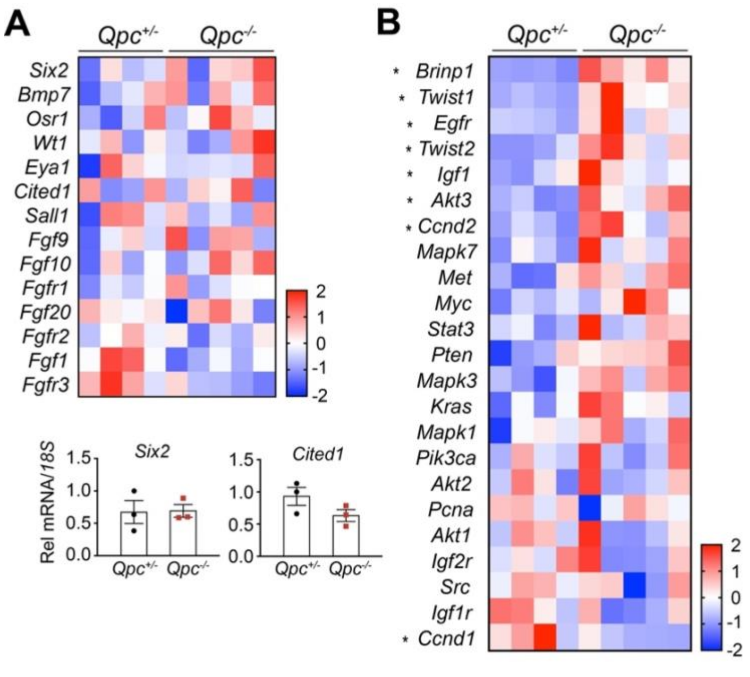

Supplementary Figure S7 (associated with Figure 6). Growth factor-activated signaling pathways are not downregulated in *Six2-Qpc*$^{-/-}$ kidneys. (A) Relative expression levels of cap mesenchyme-associated genes in SIX2 progenitor cells isolated by FACS from heterozygous *Six2-Qpc*$^{+/-}$ control and *Six2-Qpc*$^{-/-}$ kidneys at E18.5; *Six2* and *Cited1* transcript levels were analyzed by qPCR. (B) Relative expression levels of growth-related genes in SIX2 progenitor cells isolated by FACS from heterozygous *Six2-Qpc*$^{+/-}$ control and *Six2-Qpc*$^{-/-}$ kidneys at E18.5; heat maps were generated by RNA-seq analysis. Data are presented as mean ± SEM; Student's *t*-test, 2-tailed Asterisks indicate statistically significant differences

Supplementary Figure S8 (associated with Figure 7). Purine and pyrimidine metabolites are reduced in *Six2-Qpc*$^{-/-}$ kidneys. Relative abundance of purine and pyrimidine metabolites in *Cre*$^{-}$ littermate control and *Six2-Qpc*$^{-/-}$ kidneys assessed by mass spectrometry at age P0. Data are presented as mean ± SEM; Student's *t*-test; 2-tailed, *P<0.05.

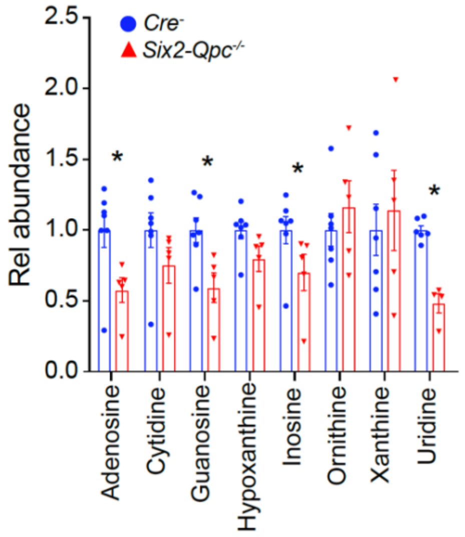

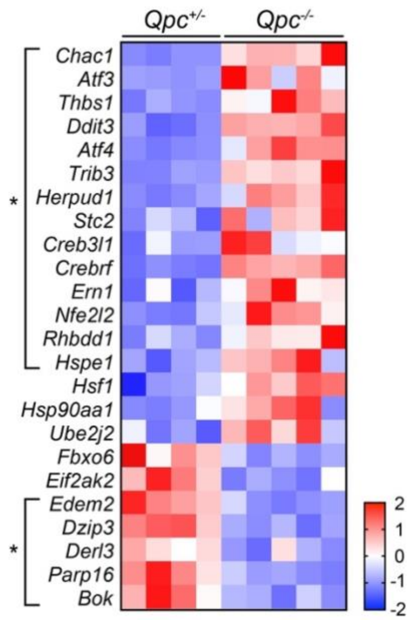

Supplementary Figure S9 (associated with Figure 7). Activation of activating transcription factor 4 (ATF4)-mediated stress responses in *Six2-Qpc*$^{-/-}$ kidneys. Increased expression of genes involved in the integrated stress response. SIX2 progenitor cells were isolated by FACS from heterozygous *Six2-Qpc*$^{+/-}$ control and *Six2-Qpc*$^{-/-}$ kidneys at age E18.5; heat map was generated by RNA-seq. Asterisks indicate statistically significant differences.

4. REFERENCES

1. Liu Q, Davidoff O, Niss K*, et al.* Hypoxia-inducible factor regulates hepcidin via erythropoietin-induced erythropoiesis. *J Clin Invest* 2012; **122:** 4635-4644.
2. Kobayashi H, Liu Q, Binns TC*, et al.* Distinct subpopulations of FOXD1 stroma-derived cells regulate renal erythropoietin. *J Clin Invest* 2016; **126:** 1926-1938.
3. Kobayashi H, Liu J, Urrutia AA*, et al.* Hypoxia-inducible factor prolyl-4-hydroxylation in FOXD1 lineage cells is essential for normal kidney development. *Kidney Int* 2017; **92:** 1370-1383.
4. Barak H, Boyle SC. Organ culture and immunostaining of mouse embryonic kidneys. *Cold Spring Harb Protoc* 2011; **2011:** pdb.prot5558.
5. Patel DP, Krausz KW, Xie C*, et al.* Metabolic profiling by gas chromatography-mass spectrometry of energy metabolism in high-fat diet-fed obese mice. *PLoS One* 2017; **12:** e0177953.
